# Effect of body mass index on survival of patients with stage I non-small cell lung cancer

**DOI:** 10.1186/s40880-016-0170-7

**Published:** 2017-01-10

**Authors:** Hao-Jun Xie, Xu Zhang, Zhen-Qiang Wei, Hao Long, Tie-Hua Rong, Xiao-Dong Su

**Affiliations:** 1 Department of Thoracic Surgery, State Key Laboratory of Oncology in Southern China, Collaborative Innovation Center for Cancer Medicine, Sun Yat-Sen University Cancer Center, 651 Dongfeng Rd. East, Guangzhou, 510060 Guangdong P. R. China; 2 Lung Cancer Institute, Sun Yat-Sen University, Guangzhou, 510060 Guangdong P. R. China

**Keywords:** Non-small cell lung cancer, Early stage, Body mass index, Survival, Surgery

## Abstract

**Background:**

Body mass index (BMI) has a U-shaped association with lung cancer risk. However, the effect of BMI on prognosis is controversial. This retrospective study aimed to investigate the effect of BMI on the survival of patients with stage I non-small cell lung cancer (NSCLC) after surgical resection.

**Methods:**

In total, 624 consecutive stage I NSCLC patients who underwent radical resection were classified into four groups according to their BMI: underweight (BMI < 18.5 kg/m^2^), normal weight (BMI = 18.5–22.4 kg/m^2^), overweight (BMI = 22.5–28.0 kg/m^2^), and obese (BMI > 28.0 kg/m^2^). The effect of BMI on progression-free survival (PFS) and overall survival (OS) was estimated using the Kaplan–Meier method and Cox proportional hazards model. Postoperative complications in each group were analyzed using the Chi square test or Fisher’s exact test.

**Results:**

A univariate analysis showed that PFS and OS were longer in the overweight group than in other groups (both *P* < 0.05). A multivariate analysis showed that OS was longer in the overweight group than in other groups (compared with the other three groups in combination: hazard ratio [HR] = 1.87, 95% confidence interval [CI] 1.30–2.68, *P* = 0.003; compared with the underweight group: HR = 2.24, 95% CI 1.18–4.25, *P* = 0.013; compared with the normal weight group: HR = 1.58, 95% CI 1.07–2.33, *P* = 0.022; compared with the obese group: HR = 2.87, 95% CI 1.48–5.59, *P* = 0.002), but PFS was similar among the groups (HR = 1.28, 95% CI 0.97–1.68, *P* = 0.080). A subgroup analysis showed an association between being overweight and prolonged OS in patients at stage T1a (*P* = 0.024), T1b (*P* = 0.051), and T2a (*P* = 0.02), as well as in patients with a non-smoking history (*P* = 0.001). Overweight patients had lower rates of postoperative complications, such as respiratory failure (compared with the underweight and obese groups: *P* = 0.014), myocardial infarction (compared with the obese group: *P* = 0.033), and perioperative death (compared with the other three groups: *P* = 0.016).

**Conclusions:**

Preoperative BMI is an independent prognostic factor for stage I NSCLC patients after resection, with overweight patients having a favorable prognosis.

## Background

The global incidence and mortality of lung cancer are the highest among all cancers. Furthermore, lung cancer is responsible for numerous public health problems [1–3]. Despite advances in surgical techniques and the incorporation of new therapeutic approaches, the 5-year survival rate for lung cancer patients has remained low [1, 4].

Strong evidence indicates that nutritional status, including weight and diet, might affect long-term survival of patients with certain types of cancer [5, 6]. The association between the body mass index (BMI) and the risk of lung cancer has been well established [7–12]. Some studies have shown that lung cancer mortality is lower in obese patients [13–16], but these studies have lacked details, such as histological type, disease stage, and treatment modality. Other studies, which only focused on advanced non-small cell lung cancer (NSCLC), showed that a lower BMI was related to a higher mortality [17]. An association between BMI and the survival of patients with operable early-stage NSCLC has not yet been definitively demonstrated. This association could help surgeons identify patients at high risk of recurrence or death.

Directing the treatment and accurately predicting the prognosis of early-stage NSCLC patients are important, as is increasing public awareness of the role lifestyle plays in cancer survivorship. The World Health Organization has recommended BMI cutoff points for underweight, normal weight, overweight, and obese, which are calculated to predict health risks, including the risks for all types of cancer and non-cancer disease [18, 19]. However, whether these cutoff points are suitable for the Asian population has remained a matter of debate [20]. In two large cohort studies that examined the association between BMI and lung cancer risk in the Chinese population, Yang et al. [13] used the BMI cutoff points of 18.5, 20.0, 22.4, and 25.0 kg/m^2^ for underweight, normal weight, overweight, and obese, respectively, whereas Koh et al. [21] proposed the cutoff points of 20.0, 24.0, and 28.0 kg/m^2^ for normal weight, overweight, and obese, respectively.

Adapting the BMI cutoff points proposed in Asian studies [13, 21], we analyzed a large cohort of Chinese patients with stage I NSCLC who underwent complete surgical resection to investigate the association between BMI and the survival of patients with stage I NSCLC, as well as complications after resection.

## Patients and methods

### Patient selection

This study was officially approved by the ethics committee of Sun Yat-sen University Cancer Center. We identified consecutive patients with stage I NSCLC (according to the 7th edition TNM classification) [22] who underwent complete surgical resection at Sun Yat-sen University Cancer Center between December 2005 and December 2010. Patients were excluded if they had received neoadjuvant chemotherapy, had an unknown BMI, had a history of other types of cancer, or had residual tumor tissue after surgery. Patient characteristics and postoperative complications were analyzed. All of the patients underwent a lobectomy, bilobectomy, or pneumonectomy with complete lymph node dissection through the surgical approaches of open thoracotomy or video-assisted thoracoscopy.

### BMI

BMI was calculated based on direct height and weight measurements before treatment. Specific BMI cutoff points for the Asian population have yet to be defined [18–20]. Therefore, we modified the BMI cutoff points proposed by Yang et al. [13] (18.5, 20.0, 22.4, and 25.0 kg/m^2^) and Koh et al. [21] (20, 24, and 28 kg/m^2^) and divided the patients into four groups: underweight (BMI < 18.5 kg/m^2^), normal weight (BMI = 18.5–22.4 kg/m^2^), overweight (BMI = 22.5–28.0 kg/m^2^), and obese (BMI > 28.0 kg/m^2^).

### Definitions of postoperative complications

All complications between the time of surgery and hospital discharge were documented. Respiratory failure was defined as the requirement for postoperative mechanical ventilation longer than 24 h. Postoperative pyrexia was defined as a body temperature ≥38°C for a period >24 h following surgery. Chylothorax was defined as elevated triglyceride levels in milky drained fluid. Postoperative hemorrhage required reoperation. Myocardial infarction and arrhythmia were detected by electrocardiogram. Pneumothorax was detected by clinical observation and chest X-ray radiography. Atelectasis was detected by clinical observation, chest X-ray radiography, and fiberoptic bronchoscopy. Perioperative death was defined as death within 1 month after surgery.

### Follow-up

Generally, the patients were followed every 3 months for the first year and twice a year thereafter. However, follow-up interval was shortened for patients with specific symptoms. Regular follow-up examinations included a physical examination, blood chemistry analysis, tumor marker measurement, and computed tomography scan or chest X-ray radiography. The follow-up duration was calculated from the date of surgery to the date of the event or the last contact. Follow-up was continued until May 2015.

### Statistical analysis

Statistical analyses were performed using SPSS 22.0 for Windows software (SPSS Inc., Chicago, IL, USA). Differences among the four BMI groups were tested using the Kruskal–Wallis test. The associations between BMI and clinicopathologic parameters or postoperative complications were analyzed using the Chi square test or Fisher’s exact test. Progression-free survival (PFS) was calculated from the date of surgery to the date of tumor recurrence, metastasis, or death from any cause. Overall survival (OS) was calculated from the date of surgery to the date of death from any cause. Survival curves were plotted using the Kaplan–Meier method and analyzed using the log-rank test. A multivariate analysis was performed using the Cox proportional hazards regression model with the backward stepwise procedure (the entry and removal probabilities were 0.05 and 0.10). A significant difference was declared if the *P* value from a two-tailed test was <0.05.

## Results

### Patient characteristics by BMI

Seven hundred consecutive patients with stage I NSCLC underwent complete surgical resection between December 2005 and December 2010. Among them, 624 patients were included in the study and were divided into four groups according to BMI. The other 76 patients were excluded because they had received neoadjuvant chemotherapy (17 patients), had an unknown BMI (3 patients), had a history of other types of cancer (50 patients), had residual tumor tissue after surgery (4 patients), or a combination of the above (2 patients).

The patient characteristics are shown in Table 1. As compared with a lower BMI, a higher BMI was associated with higher rates of hypertension (*P* < 0.001), smoking (*P* = 0.001), and high preoperative forced expiratory volume in 1 s (FEV1)/forced vital capacity (FVC) ratios (*P* = 0.011). Normal weight patients were more likely to have visceral pleural invasion (*P* = 0.005) and to have a higher T stage (*P* < 0.001) and TNM stage (*P* < 0.001) than underweight, overweight, and obese patients.

**Table 1:** The baseline clinicopathologic characteristics of underweight, normal weight, overweight, and obese patients with stage I NSCLC, stratified by BMI

Characteristic	Overall (cases)	Underweight [cases (%)]	Normal weight [cases (%)]	Overweight [cases (%)]	Obese [cases (%)]	*P* value
Total	624	44	245	306	29	
Gender						0.668
Male	408 (65.4)	26 (13.6)	166 (67.8)	198 (64.7)	18 (62.1)	
Female	216 (34.6)	18 (86.4)	79 (32.2)	108 (35.3)	11 (37.9)	
Age (years)						0.991
<60	285 (45.7)	20 (2.3)	114 (46.5)	138 (45.1)	13 (44.8)	
≥60	339 (54.3)	24 (97.7)	131 (53.5)	168 (54.9)	16 (55.2)	
Smoking						0.001
Never	318 (51.0)	23 (4.5)	101 (41.2)	178 (58.2)	16 (55.2)	
Current or ever	306 (49.0)	21 (95.5)	144 (58.8)	128 (41.8)	13 (44.8)	
Hypertension						<0.001
With	150 (24.0)	6 (13.6)	38 (15.5)	93 (30.4)	13 (44.8)	
Without	474 (76.0)	38 (86.4)	207 (84.5)	213 (69.6)	16 (55.2)	
Diabetes						0.318
With	46 (7.4)	1 (2.3)	18 (7.3)	23 (7.5)	4 (13.8)	
Without	578 (92.6)	43 (97.7)	227 (92.7)	283 (92.5)	25 (86.2)	
Heart disease						0.482
With	18 (2.9)	2 (4.5)	7 (2.9)	7 (2.3)	2 (6.9)	
Without	606 (97.1)	42 (95.5)	238 (97.1)	299 (97.7)	27 (93.1)	
FEV1/FVC (%)						0.011
<70	83 (13.3)	11 (25.0)	40 (16.3)	30 (9.8)	2 (6.9)	
≥70	541 (86.7)	33 (75.0)	205 (83.7)	276 (90.2)	27 (93.1)	
Type of surgery						0.979
Open thoracotomy	537 (86.1)	37 (84.1)	210 (85.7)	264 (86.3)	25 (86.2)	
VATS	87 (13.9)	7 (15.9)	35 (14.3)	42(13.7)	4 (13.8)	
Extent of resection						0.681
Lobectomy or bilobectomy	611 (97.9)	44 (100.0)	239 (97.6)	299 (97.7)	29 (100.0)	
Pneumonectomy	13 (2.1)	0 (0.0)	6 (2.4)	7 (2.3)	0 (0.0)	
Adjuvant chemotherapy						0.967
Absent	486 (77.9)	34 (77.3)	193 (78.8)	236 (77.1)	23 (79.3)	
Present	138 (22.1)	10 (22.7)	52 (21.2)	70 (22.9)	6 (20.7)	
Histological type						0.432
Adenocarcinoma	406 (65.1)	29 (65.9)	152 (62.0)	203 (66.3)	22 (75.9)	
Squamous cell carcinoma	140 (22.4)	12 (27.3)	62 (25.3)	63 (20.6)	3 (10.3)	
Other NSCLC	78 (12.5)	3 (6.8)	31 (12.7)	40 (13.1)	4 (13.8)	
Histologic differentiation						0.131
Poor	212 (34.0)	17 (38.6)	94 (38.4)	92 (30.1)	9 (31.0)	
Moderate	300 (48.1)	17 (38.6)	113 (46.1)	159 (52.0)	11 (37.9)	
Well	112 (17.9)	10 (22.7)	38 (15.5)	55 (18.0)	9 (31.0)	
Visceral pleural invasion						0.005
Absent	408 (65.4)	32 (72.7)	140 (57.1)	212 (69.3)	23 (79.3)	
Present	216 (34.6)	12 (27.3)	105 (42.9)	94 (30.7)	6 (20.7)	
T stage						<0.001
T1a	132 (21.2)	10 (22.7)	36 (14.7)	78 (25.5)	8 (27.6)	
T1b	113 (18.1)	13 (29.5)	31 (12.7)	59 (19.3)	9 (31.0)	
T2a	379 (60.7)	21 (47.7)	178 (72.7)	169 (55.2)	12 (41.4)	
Clinical TNM stage						<0.001
IA	245 (39.3)	23 (52.3)	67 (27.3)	137 (44.8)	17 (58.6)	
IB	379 (60.7)	21 (47.7)	178 (72.7)	169 (55.2)	12 (41.4)	

Underweight BMI < 18.5 kg/m^2^; normal weight BMI 18.5–22.4 kg/m^2^; overweight BMI = 22.5–28.0 kg/m^2^; obese BMI > 28.0 kg/m^2^

*NSCLC* non-small cell lung cancer, *BMI* body mass index, *FEV1/FVC* forced expiratory volume in 1 s/forced vital capacity, *VATS* video-assisted thoracic surgery

Overweight and normal weight patients were less likely to experience postoperative respiratory failure (*P* = 0.014) than underweight and obese patients, whereas obese patients were more likely to have myocardial infarction after surgery (*P* = 0.033) than underweight, normal weight, and overweight patients. Furthermore, perioperative death was significantly less common in the overweight group than in other groups (*P* = 0.016, Table 2).

**Table 2:** Postoperative complications of underweight, normal weight, overweight, and obese patients with stage I NSCLC

Characteristic	Overall (cases)	Underweight [cases (%)]	Normal weight [cases (%)]	Overweight [cases (%)]	Obese [cases (%)]	*P* value
Total	624	44	245	306	29	
Respiratory failure						0.014
Absent	605 (97.0)	40 (90.9)	240 (98.0)	299 (97.7)	26 (89.7)	
Present	19 (3.0)	4 (9.1)	5 (2.0)	7 (2.3)	3 (10.3)	
Pyrexia						0.965
Absent	502 (80.4)	36 (81.8)	195 (79.6)	247 (80.7)	24 (82.8)	
Present	122 (19.6)	8 (18.2)	50 (20.4)	59 (19.3)	5 (17.2)	
Chylothorax						0.339
Absent	616 (98.7)	43 (97.7)	241 (98.4)	304 (99.3)	28 (96.6)	
Present	8 (1.3)	1 (2.3)	4 (1.6)	2 (0.7)	1 (3.4)	
Hemorrhage						0.074
Absent	612 (98.1)	44 (100.0)	236 (96.3)	303 (99.0)	29 (100.0)	
Present	12 (1.9)	0 (0.0)	9 (3.7)	3 (1.0)	0 (0.0)	
Myocardial infarction						0.033
Absent	618 (99.0)	44 (100.0)	243 (99.2)	304 (99.3)	27 (93.1)	
Present	6 (1.0)	0 (0.0)	2 (0.8)	2 (0.7)	2 (6.9)	
Arrhythmia						0.484
Absent	559 (89.6)	38 (86.4)	225 (91.8)	270 (88.2)	26 (89.7)	
Present	65 (10.4)	6 (13.6)	20 (8.2)	36 (11.8)	3 (10.3)	
Pneumothorax						0.100
Absent	473 (75.8)	29 (65.9)	179 (73.1)	240 (78.4)	25 (86.2)	
Present	151 (24.2)	15 (34.1)	66 (26.9)	66 (21.6)	4 (13.8)	
Atelectasis						0.818
Absent	579 (92.8)	40 (90.9)	228 (93.1)	283 (92.5)	28 (96.6)	
Present	45 (7.2)	4 (9.1)	17 (6.9)	23 (7.5)	1 (3.4)	
Death						0.016
Absent	614 (98.4)	42 (95.5)	240 (98.0)	305 (99.7)	27 (93.1)	
Present	10 (1.6)	2 (4.5)	5 (2.0)	1 (0.3)	2 (6.9)	

### Univariate and multivariate analyses

**Fig. 1:**
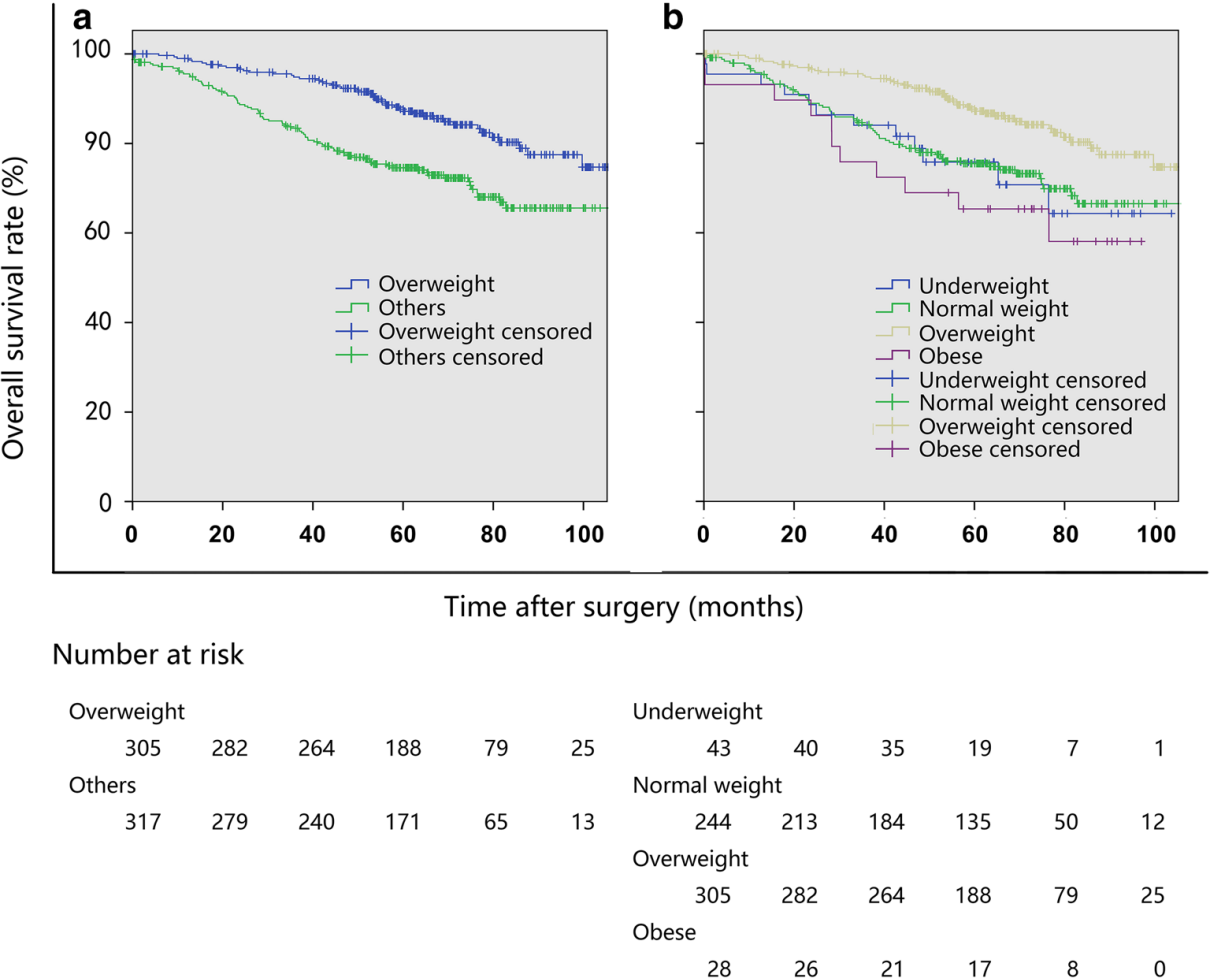
Kaplan–Meier overall survival (OS) curves of patients with stage I non-small cell lung cancer (NSCLC) stratified by body mass index (BMI). Underweight BMI < 18.5 kg/m^2^; normal weight BMI 18.5–22.4 kg/m^2^; overweight BMI 22.5–28.0 kg/m^2^; and obese: BMI > 28.0 kg/m^2^. **a** The OS was longer in the overweight group than in other groups in combination (*P* = 0.001). **b** The OS was longer in the overweight group than in the underweight group (*P* = 0.012), the normal weight group (*P* = 0.001), and the obese group (*P* = 0.002)

**Fig. 2:**
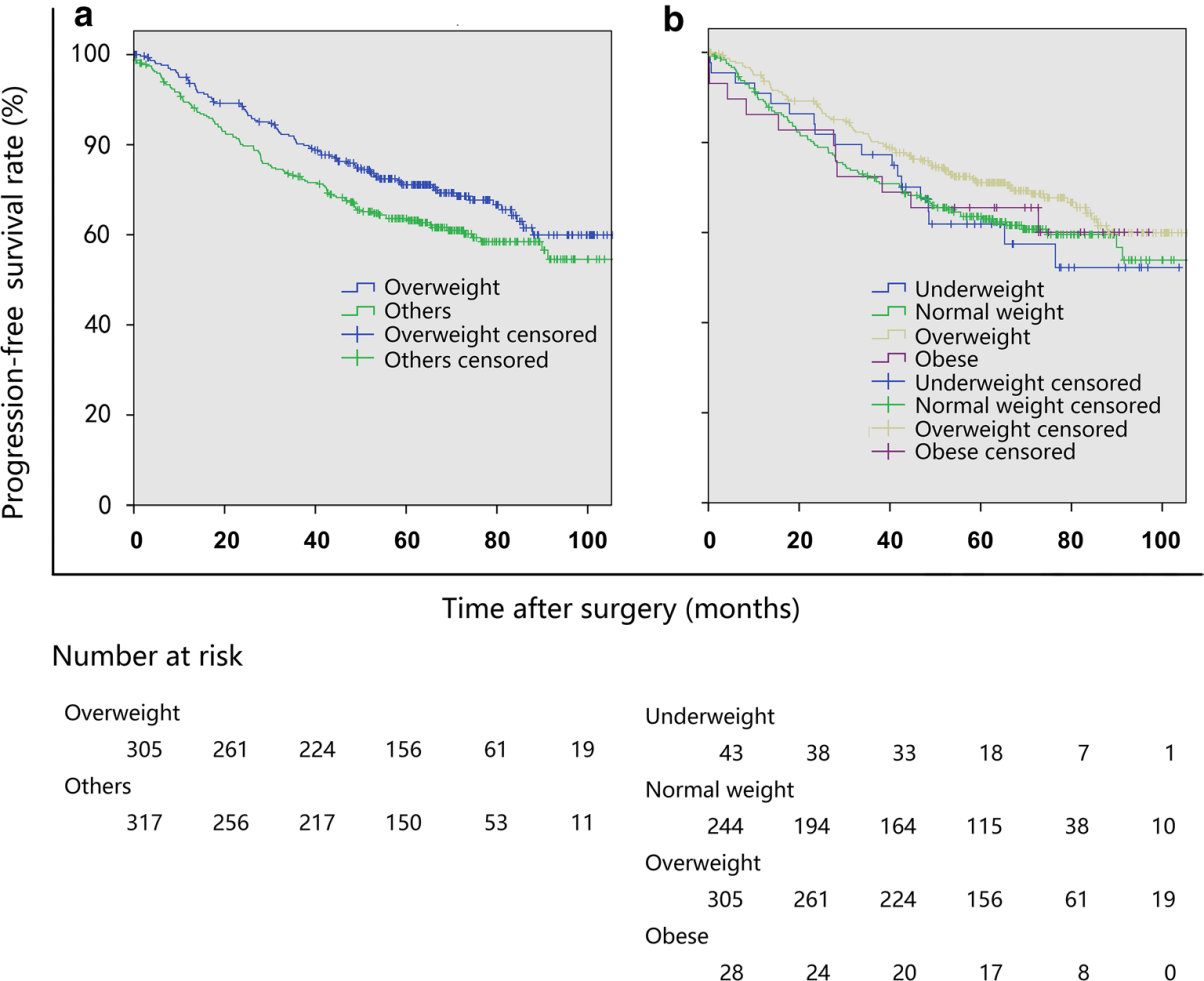
Kaplan–Meier progression-free survival (PFS) curves of patients with stage I NSCLC stratified by BMI. **a** The PFS was longer in the overweight group than in other groups in combination (*P* = 0.034). **b** The PFS was longer in the overweight group than in the normal weight group (*P* = 0.045), but differences were not significant between the overweight group and the underweight group (*P* = 0.172) and the obese group (*P* = 0.514)

The median follow-up duration of the entire cohort was 63.2 months (range 47.8–78.1 months). The univariate survival analysis showed significant differences in PFS and OS between the overweight group and other groups. The OS of overweight patients was significantly longer than those of underweight, normal weight, and obese patients (*P* = 0.001; Table 3; Fig. 1). The PFS of overweight patients was significantly longer than those of patients in other groups (*P* = 0.034, Table 4; Fig. 2). The multivariate survival analysis showed that BMI was an independent factor associated with OS, and that overweight patients had a significantly lower risk of death from stage I NSCLC after surgery than did patients in other groups (compared with the underweight group: hazard ratio [HR] = 2.24, 95% confidence interval [CI] 1.18–4.25, *P* = 0.013; compared with the normal weight group: HR = 1.58, 95% CI 1.07–2.33, *P* = 0.022; compared with the obese group: HR = 2.87, 95% CI 1.48–5.59, *P* = 0.002). Other independent factors that might affect OS included age, smoking history, extent of resection, histological differentiation, and T stage. Independent factors that were associated with PFS included age, smoking, extent of resection, adjuvant chemotherapy, BMI, and FEV1/FVC ratio.

**Table 3:** Univariate and multivariate overall survival (OS) analyses for patients with stage I NSCLC

Variate	OS rate (%)		Univariate analysis *P* value	Multivariate analysis	
	3-year	5-year		HR (95% CI)	*P* value
Age (years)			0.002	1.83 (1.26–2.64)	0.001
<60	92.6 ± 1.6	85.6 ± 2.2			
≥60	85.8 ± 1.9	77.1 ± 2.4			
Smoking			<0.001	1.45 (1.05–2.10)	0.047
Never	94.1 ± 1.3	86.4 ± 2.0			
Current or ever	83.4 ± 2.2	75.1 ± 2.6			
BMI			0.001		0.003
Underweight	84.0 ± 5.5	75.8 ± 6.7	0.012	2.24 (1.18–4.25)	0.013
Normal weight	84.2 ± 2.4	75.5 ± 2.9	<0.001	1.58 (1.07–2.33)	0.022
Overweight	94.8 ± 1.3	87.7 ± 2.0	Reference	Reference	Reference
Obese	75.9 ± 7.9	65.3 ± 8.9	0.002	2.87 (1.48–5.59)	0.002
Extent of resection			<0.001	5.01 (2.26–11.07)	<0.001
Lobectomy or bilobectomy	89.6 ± 1.3	81.6 ± 1.6			
Pneumonectomy	51.3 ± 15.8	41.0 ± 15.6			
Histological differentiation			0.001	0.76 (0.58–0.99)	0.039
Poor	83.0 ± 2.7	73.5 ± 3.2			
Moderate	91.5 ± 1.6	84.0 ± 2.2			
Well	93.2 ± 2.5	86.7 ± 3.5			
T stage			0.005	1.40 (1.08–1.80)	0.011
T1a	93.8 ± 2.1	90.4 ± 2.7			
T1b	89.0 ± 3.0	82.0 ± 3.8			
T2a	87.3 ± 1.8	77.3 ± 2.3			

The data of survival rates are expressed as mean ± standard error

*HR* hazard ratio, *CI* confidence interval

**Table 4:** Univariate and multivariate progression-free survival (PFS) analyses for patients with stage I NSCLC

Variate	PFS rate (%)		Univariate analysis *P* value	Multivariate analysis	
	3-year	5-year		HR (95% CI)	*P* value
Age (years)			0.068	1.45 (1.09–1.93)	0.011
<60	79.4 ± 2.4	69.9 ± 2.8			
≥60	73.9 ± 2.4	65.0 ± 2.7			
Smoking			0.003	1.43 (1.08–1.90)	0.014
Never	80.8 ± 2.2	72.2 ± 2.6			
Current or ever	71.9 ± 2.6	62.0 ± 2.9			
BMI			0.034	1.28 (0.97–1.68)	0.080
Overweight	80.2 ± 2.3	71.1 ± 2.7			
Others	72.9 ± 2.5	63.6 ± 2.8			
Extent of resection			<0.001	4.71 (2.23–9.97)	<0.001
Lobectomy or bilobectomy	77.2 ± 1.7	68.0 ± 2.0			
Pneumonectomy	38.4 ± 14.7	28.8 ± 13.8			
FEV1/FVC (%)			0.910	0.70 (0.46–1.07)	0.096
<70	76.3 ± 1.9	67.3 ± 2.1			
≥70	77.6 ± 4.7	66.8 ± 5.4			
Adjuvant chemotherapy			0.128	1.39 (1.01–1.91)	0.043
Absent	77.6 ± 1.9	68.7 ± 2.2			
Present	72.3 ± 3.9	61.8 ± 4.4			

The data of survival rates are expressed as mean ± standard error. Abbreviations as in Tables 1 and 3

### Subgroup analysis

**Fig. 3:**
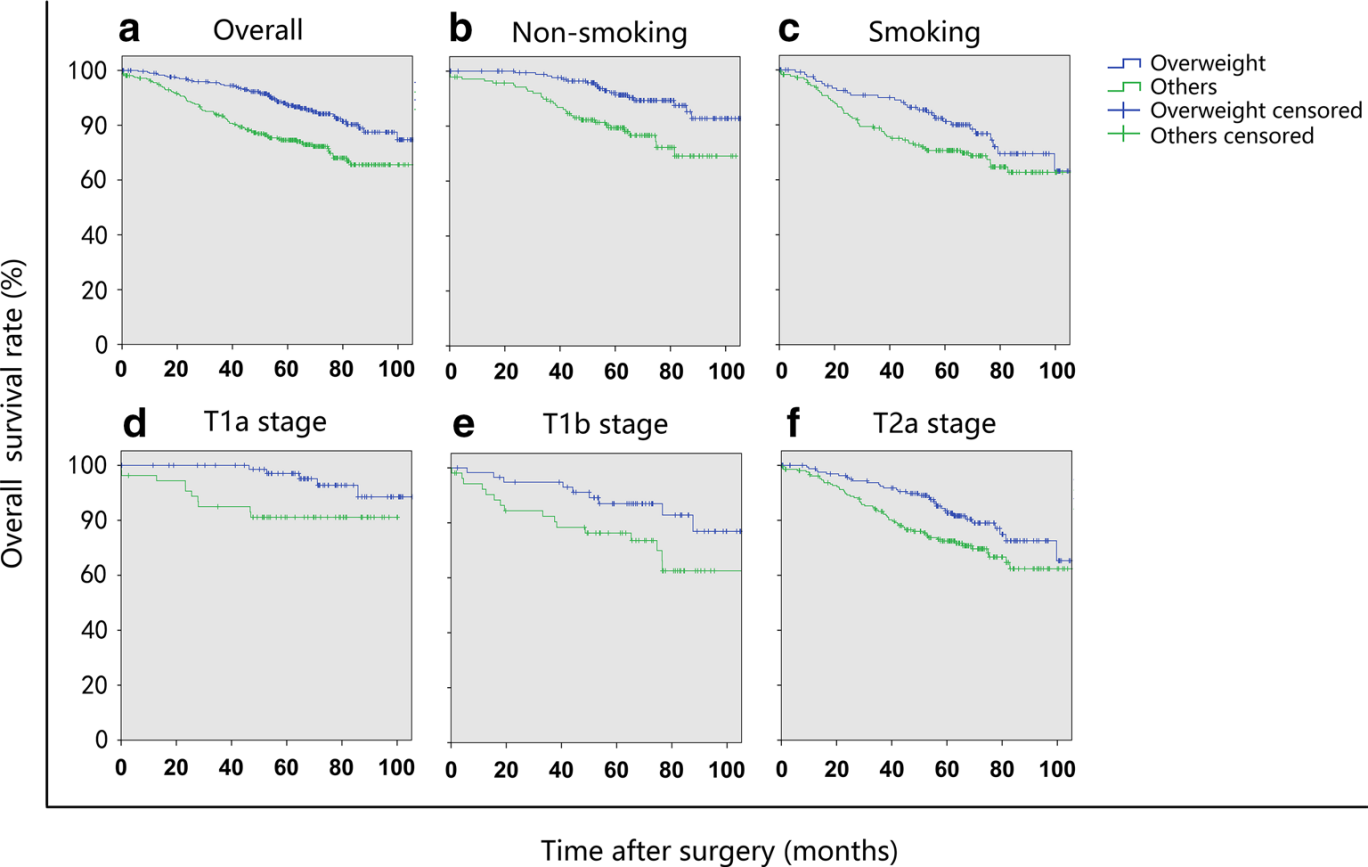
Kaplan–Meier OS curves of stage I NSCLC patients in the overweight group and other groups in combination stratified by smoking status and T stage. **a** The OS was longer in the overweight group than in other groups in combination (*P* = 0.001); **b** in non-smoking patients, the OS was longer in the overweight group than in other groups in combination (*P* = 0.001); **c** in smoking patients, the difference in OS was insignificant between the overweight group and other groups in combination (*P* = 0.083); **d** in patients with T1a stage NSCLC, the OS was longer in the overweight group than in other groups in combination (*P* = 0.024); **e** in patients with T1b stage NSCLC, the difference in OS was insignificant between the overweight group and other groups in combination (*P* = 0.051); and **f** in patients with T2a stage NSCLC, the OS of the overweight group was longer than that of other groups in combination (*P* = 0.020)

The univariate survival analyses showed that when stratified by smoking status and T stage, the association between being overweight and prolonged OS still existed (Table 5; Fig. 3).

**Table 5:** Subgroup OS analysis for stage I NSCLC patients in the overweight group and other groups, stratified by smoking status and T stage

Prognostic factor	OS rate (%)		Univariate analysis	
	3-year	5-year	*P* value (log-rank)	*P* value (Breslow)
Non-smoking			0.001	<0.001
Overweight	98.2 ± 1.0	92.1 ± 2.2		
Others	89.1 ± 2.7	79.4 ± 3.6		
Smoking			0.083	0.024
Overweight	89.9 ± 2.8	81.2 ± 3.7		
Others	78.8 ± 3.1	70.7 ± 3.5		
T1a stage			0.024	0.004
Overweight	100.0 ± 0	97.0 ± 2.1		
Others	85.0 ± 4.9	81.1 ± 5.4		
T1b stage			0.051	0.040
Overweight	94.8 ± 2.9	87.0 ± 4.6		
Others	82.4 ± 5.3	76.3 ± 6.0		
T2a stage			0.020	0.005
Overweight	92.4 ± 2.1	83.5 ± 3.1		
Others	83.2 ± 2.6	72.5 ± 3.2		

The data of survival rates are expressed as mean ± standard error

## Discussion

In our study, overweight patients showed a significantly longer OS than patients in other groups, as determined with both the univariate and multivariate survival analyses. Overweight patients also had a longer PFS than patients in other groups, according to the univariate survival analysis. Furthermore, considering the potential effect of different T stages on the outcomes, we stratified the patients by T stage and re-tested the results. Using either the log-rank test or the Breslow test, we found that overweight patients had longer OS than did patients in other groups for each T stage, indicating the prognostic value of BMI in early-stage NSCLC.

The relationship between BMI and the risk of lung cancer has been studied widely in different patient populations [7, 8, 11–13, 21]. An inverse association between BMI and lung cancer mortality was reported in current smokers [13] and non-smokers [14, 15]. However, all of these studies focused primarily on epidemiology, including all histological types or stages of lung cancer, but the treatment details were not provided. In the present study, we focused on operable stage I NSCLC and found that being overweight had a significant positive effect on the survival of stage I NSCLC patients after surgery. A low BMI (<18.5 kg/m^2^) has been shown to increase the risk of early postoperative death following en bloc chest wall and lung resection for NSCLC patients (*P* = 0.009) [23]. In addition, in a study of 640 patients after lobectomy for NSCLC, Tewari et al. [24] reported that a BMI < 18.5 kg/m^2^ (as a surrogate for impaired nutrition) was a negative predictor of long-term survival independent of tumor extension and stage. In a large cohort study examining the effect of sex on the long-term outcomes of stage I NSCLC patients after curative resection, Warwick et al. [25] also found that BMI was an independent prognostic factor (HR = 0.98, 95% CI 0.96–1.00, *P* = 0.02). Although patients were not grouped by BMI, these studies demonstrated that a low BMI was a negative prognostic factor for NSCLC after resection. This finding may partly support our finding that survival was longer in overweight patients than in underweight and normal weight patients.

The mechanism by which being overweight might prolong patient survival is still not well understood. Some reports have explained that the association between BMI and lung cancer mortality is caused by smoking history [13]. A high smoking rate was found in patients with a low BMI, and smoking has been reported to have an negative effect on survival [26, 27]. In accordance with other studies [13, 16], we found that fewer overweight and obese patients were smokers as compared with underweight and normal weight patients. However, in the subgroup analysis, we found that the significant prognosis-protective effect of being overweight persisted in non-smokers. In smokers, the overweight group had significantly longer survival than did other groups, as determined using the Breslow test, and while not significant, this group also tended to show longer survival as measured using the log-rank test. Yang et al. [14] and Parr et al. [15] demonstrated that the inverse association between BMI and mortality persisted not only in smokers but also in non-smokers. This finding indicates that BMI is an independent risk factor for lung cancer patient survival. After excluding patients who died within 3 years after diagnosis, Leung et al. [16] also found that BMI was independently and negatively associated with death from lung cancer in smokers and never-smokers. This previous evidence and the findings of our study indicate that BMI is an independent factor for survival. The mechanism of the effect of BMI on the survival of NSCLC patients requires further investigation. Brennan et al. [28] found that the fat mass- and obesity-associated (*FTO*) gene, which is linked with increased BMI [29], was associated with a decreased risk of lung cancer. This finding provides us another perspective from which to consider how BMI affects the survival of lung cancer patients, although a direct association between lung cancer patient survival and the *FTO* gene has not yet been demonstrated.

In the current study, although being overweight appeared to prolong survival in stage I NSCLC patients, the obese group had a similar outcome as the normal and underweight groups. This finding contradicts those of other studies [13, 14], in which the obese group of lung cancer patients usually had the lowest mortality. However, these studies were based on all types of pulmonary malignancies, including small cell lung cancer and advanced-stage NSCLC, which have a poor prognosis. Stage I NSCLC in particular is biased toward non-cancer-related death because patients are more likely to be cured and to have a long life expectancy. The obese patients in our study actually had higher rates of preoperative diseases, such as hypertension, as well as postoperative complications, such as respiratory failure and myocardial infarction, compared with patients in other groups. These complications might cause poor health after surgery and may lead to death early after treatment. Using data from the Asia-Pacific Cohort Studies Collaboration, Parr et al. [14] examined the association between BMI and mortality from over 20 cancer sites in adults and found a result similar to ours. They compared normal weight and obese groups and found that significantly lower lung cancer mortality persisted in the overweight group in both smokers and non-smokers. Therefore, there was a U-shaped relation between BMI and lung cancer mortality. There have been few studies on the association between BMI and operable stage I NSCLC. The number of obese patients in our study was limited. Therefore, our results must be interpreted with caution.

We acknowledge some limitations in our study. Because BMI cutoff points have yet to be defined for the Asian population, patient grouping may lead to a selection bias. Additionally, we failed to explain the mechanism of how BMI affects lung cancer patient survival. Biomarkers and genetic markers should be used in further research on this topic.

## Conclusions

Being overweight appears to be more favorable than being obese, normal weight, and underweight for the survival of patients with stage I NSCLC after complete surgical resection. Among either non-smokers or smokers, overweight patients had significantly longer survival than underweight, normal weight, and obese patients. The mechanism of how BMI affects lung cancer patient survival remains to be elucidated.
